# Supplementary material for: Circular RNA hsa_circ_0051040 Promotes Hepatocellular Carcinoma Progression by Sponging miR-569 and Regulating ITGAV Expression
Source: Cells. 2022 Nov 11;11(22):3571. doi: 10.3390/cells11223571 (PMC9688127; doi:10.3390/cells11223571)
Supplement: Supplementary file 1 [file cells-11-03571-s001.zip › cells-1956974-supplementary.pdf]

Supplementary Materials:

Table S1. The clinicopathological parameters of plasma cases.

**Characteristics**	**Normal (n=122)**	**Benign (n=24)**	**HCC (n=186)**
Gender			
Male	62	19	136
Female	60	5	50
Age（year）			
Mean (range)	39 (22-73)	54 (34-74)	57 (36-80)
Serum AFP (ng/ml)			
< 400	122	22	55
⩾400	0	2	131
ALT（U/L, mean±SD）	26.2±19.3	362.5±460.3	39.1±27.1
AST（U/L, mean±SD）	22.1±18.2	275.6±475.0	46.5±36.8
Tumor size（cm）			
＜5cm	0	0	121
⩾5cm	0	0	65
Lymph node metastasis			
Positive	0	0	48
Negative	0	0	118

Table S2. Sequences of the FISH probes used in this study.

**Gene name**	**Probe (5’-3’)**
hsa_circ_0051040 (FISH)	Cy3-ACTGCATAGACTAGACCATCGACGGATGAGTCTTTTAATA
hsa-miR-569 (FISH)	FAM-ACTTTCCAGGATTCATTAACT
18srRNA (FISH)	Cy3-CTTCCTTGGATGTGGTAGCCGTTTC

Table S3. Sequences of the qRT-PCR primers used in this study.

**Gene name**	**Forward primer (5'-3')**	**Reverse primer (5'-3')**
hsa_circ_0051040 (divergent)	TCATCCGTCGATGGTCTAGTCT	ATTGCGATGAGCATGCGGTA
hsa_circ_0051040 (convergent)	AAGGCCAACTGCATTGACTCCAC	GCTCCTGCGGCTTCATGTTCTC
ITGAV	ACAGGCAATAGAGATTATGCCA	TTTATCCTGTTTCGACCTCACA

18srRNA	GTAACCCGTTGAACCCCATT	CCATCCAATCGGTAGTAGCG
β-actin	GAGAAATCTGGCACCACACC	GGATAGCACAGCCTGGATAGCAA
GAPDH	AAGGTCGGAGTCAACGGATTTG	CCATGGGTGGAATCATATTGGAA
GAPDH (divergent)	GAAGGTGAAGGTCGAGTC	GAAGATGGTGATGGGATTTC
